# Identification of differential pharyngeal cytokine profiles during HIV infection

**Published:** 2011-01-10

**Authors:** RAPM Perera, PCS Tsang, C Miller, P Li, MP Lee, LP Samaranayake

**Affiliations:** 1 The University of Hong Kong, Hong Kong SAR; 2 University of Kentucky, Lexington, KY 40536, USA; 3 Queen Elizabeth Hospital, Hong Kong, Hong Kong SAR

## Background

Significantly higher pharyngeal shedding of Epstein-Barr virus (EBV) is observed during HIV infection. Increased EBV shedding in pharynx is not affected even during highly active antiretroviral theyrapy (HAART). EBV positive monocyte populations have been shown to carry EBV to pharyngeal mucosa. Human cytokine profiles are often altered to facilitate herpes virus infection. Thus pharyngeal cytokine profiles may influence EBV reactivation and shedding during HIV infection. Our objective was to compare 37 pharyngeal cytokine profiles of HIV-seropositive patients who were or were not receiving HAART therapy.

## Methods

120 HIV positive volunteers under HAART and 72 HIV patients not undergoing antiviral therapy were investigated. All Volunteers with oral pathologies and smoking were excluded. Pharyngeal secretions were collected using standard procedures and analyzed for 37 cytokine profiles using human cytokine profiler array panels and ELISA. Cytokine interactome maps generated using Ingenuity database.

## Results

Proteome profiler arrays demonstrated differential cytokine expression among HIV infected individual under HAART, HIV infected individuals without HAART and the healthy control. HIV group consisted of up regulated C5a, G-CSF, CXCL1, soluble ICAM1, IL-1α, IL-1β, IL1-Ra, IL-6, IL-8, IL-10, IL-12α, IL-13, IL-16, IL-25, IL-23α, IL-27, IL-32α, CXCL10, CXCL11, CCL-13, MIF, CCL3, CCL4, SERPIN-E1, CCL5, CXCL12, TNFα and soluble TREM1 and down regulated soluble sCD40, eGM-CSF, I-309, IFN-γ, IL-17A, IL-2, IL-4, IL-5. From these C5a, sTREM1, TNF alpha, CXCL12, CCL5, IL-17E, IL-23, IL-32α, IL-16, CCL3, IL-6 showed significantly higher expression levels in both HIV groups compared to healthy group (P < 0.05). TGF β1 levels in HIV patients undergoing HAART (0.5 +/-0.1 ng/ml) and without HAART (0.4 +/-0.1 ng/ml) were significantly increased when compared with the healthy control group (0.3 +/- 0.07 ng/ml) (P = 0.0001). TGF β1 levels had a significant positive correlation with higher CD4 counts in the group receiving HAART (P = 0.006). Cytokine interactome mapping revealed significantly increased immune cell trafficking in pharynx during HIV infection.

## Conclusion

Pharyngeal TGF β1 levels are significantly increased during HIV infection. As TGF β1 is a known trigger of Epstein Barr viral lytic gene promoters, may influence pharyngeal reactivation of Epstein-Barr virus. Additionally, increased tendency for immune cell trafficking may facilitate EBV positive monocyte trafficking towards pharyngeal mucosa during HIV infection.

